# Trends in the use of neuromuscular blocking agents, reversal agents and neuromuscular transmission monitoring: a single-centre retrospective cohort study

**DOI:** 10.1186/s13741-024-00382-y

**Published:** 2024-03-27

**Authors:** Piet Krijtenburg, Arjen de Boer, Lori D. Bash, Gert Jan Scheffer, Christiaan Keijzer, Michiel C. Warlé

**Affiliations:** 1 Department of Anesthesiology, Radboudumc, Nijmegen, the Netherlands; 2 Department of Surgery, Radboudumc, Nijmegen, the Netherlands; 3 Centre for Observational and Real-World Evidence (CORE), Merck & Co., Inc., Kenilworth, New Jersey USA

**Keywords:** Neuromuscular transmission (NMT) monitoring, Neuromuscular blocking agents (NMBAs), Reversal agents for NMBAs

## Abstract

**Background:**

Residual neuromuscular blockade (rNMB) remains a persistent and preventable problem, with serious risks.

**Methods:**

Our objective was to describe and assess patterns in the use of neuromuscular blocking agents (NMBAs), neuromuscular transmission (NMT) monitoring, and factors associated with the use of sugammadex. We performed a retrospective, observational cohort study based on electronic medical records in a large teaching hospital in the Netherlands that introduced an integrated NMT monitoring module with automatic recording in 2017. A total of 22,000 cases were randomly selected from all surgeries between January 2015 and December 2019 that required endotracheal intubation with the use of an NMBA. A total of 14,592 cases fulfilled all the inclusion criteria for complete analyses.

**Results:**

Relative NMBA usage remained the same over time. For rocuronium, spontaneous reversal decreased from 86 to 81%, sugammadex reversal increased from 12 to 18%. There was a decline in patients extubated in the operating room (OR) with neither documented NMT monitoring nor sugammadex-mediated reversal from 46 to 31%. The percentage of patients extubated in the OR without a documented train-of-four ratio ≥ 0.9, decreased from 77 to 56%. Several factors were independently associated with the use of sugammadex, including BMI > 30 kg/m^2^ (odds ratio: 1.41; 95% CI: 1.24–1.60), ASA class 3 or 4 (1.20; 1.07–1.34), age > 60 years (1.37; 1.23–1.53), duration of surgery < 120 min (3.01; 2.68–3.38), emergency surgery (1.83; 1.60–2.09), laparoscopic surgery (2.01; 1.71–2.36), open abdominal/thoracic surgery (1.56; 1.38–1.78), NMT monitoring used (5.31; 4.63–6.08), total dose of rocuronium (1.99; 1.76–2.25), and (inversely) use of inhalational anaesthetics (0.88; 0.79–0.99).

**Conclusion:**

Our data demonstrate that the implementation of NMT monitoring with automatic recording coincides with a gradual increase in the (documented) use of NMT monitoring and an increased use of sugammadex with a more precise dose. Factors associated with sugammadex use include higher age, ASA score, BMI, abdominal and thoracic surgery, higher rocuronium doses, emergency surgery and the use of NMT monitoring.

Trial registration

N/A.

**Key points:**

• Introduction of NMT monitoring with automatic recording coincides with an increase in (documented) use of NMT monitoring.

• Sugammadex is more frequently used in patients with a presumed higher a priori risk of pulmonary complications.

• Despite increased NMT monitoring and use of sugammadex a significant percentage of patients remain at potential risk of rNMB.

## Background and introduction

Since 1943, neuromuscular blocking agents (NMBAs) have been established as muscle relaxants in the practice of anaesthesia and surgery (Bowman 2006) to improve the safety of anaesthesia and later to facilitate intubation, mechanical ventilation, and surgical conditions. Currently, especially in laparoscopic surgery, increasing evidence indicates that high-dose NMBAs improve surgical conditions (Bruintjes et al. 2017). Despite advances in technology, pharmacology, and quality assurance, residual neuromuscular blockade (rNMB) remains a persistent problem. In 1979, rNMB was defined as a train-of-four ratio (TOFR) < 0.7 in the post-anaesthesia care unit (PACU) (Viby-Mogensen et al. 1979). Increasing insight into rNMB resulted in higher thresholds, from a TOFR > 0.8 in 1996 (Viby-Mogensen et al. 1996) to a TOFR > 0.9 in 2000 (Viby-Mogensen 2000). In 2003 (Eikermann et al. 2003), clinically relevant signs of rNMB were found up to a TOFR of 1.0. rNMB is iatrogenic and preventable and is associated with an increased risk of postoperative airway obstruction, pulmonary complications, and unplanned ICU admission (Berg et al. 1997; Eikermann et al. 2007; Cammu 2020; Grabitz et al. 2019; Xará et al. 2015; Blobner et al. 2020). The introduction and increased use of sugammadex, a selective binding agent, for the reversal of the non-depolarizing NMBAs rocuronium and vecuronium may have a profound effect on the incidence of rNMB. However, possible cost implications restrict the universal use of sugammadex (Watts et al. 2012). This study aims to describe and assess patterns in the use of NMBAs, reversal agents, and neuromuscular transmission (NMT) monitoring, and to identify patient-, procedure-, and anaesthesia-related factors associated with NMB management, specifically pharmacologic reversal with sugammadex. An integrated NMT module employing quantitative acceleromyography was implemented during the period under investigation. We hypothesised that this technological advancement would enhance the frequency of NMT monitoring and potentially influence the utilisation of reversal agents.

## Methods

This is a retrospective observational study using our electronic medical record (EMR), Epic (Epic Systems Corporation, Verona, WI, USA). Epic was introduced in 2013 in our hospital, Radboud University Medical Centre (Radboud UMC), an academic, large teaching hospital in Nijmegen, The Netherlands.

### Ethics

Ethical approval for this study (Ethical Committee N° 2022-13646) was provided by the Medical Research Ethical Committee Oost-Nederland, Philips van Leydenlaan 25, Nijmegen, the Netherlands (Chairperson Prof. Dr. P.N.R. Dekhuijzen,) on 22 March 2022. Written patient informed consent was waived for this study because there were no care interventions and no direct patient identifiers were used for analysis. However, surgical cases were checked if the data could be used for research purposes by opt-out consent for the use of medical data for scientific purposes registered in the EMR.

### Patients

For this study, we selected all surgical procedures between January 1, 2015, and December 31, 2019, under general anaesthesia with endotracheal intubation using a neuromuscular blocking agent (NMBA) with a minimum age of 18 years at the time of surgery, resulting in 40,240 cases. From these surgical cases, we randomly selected 22,000 cases based on our sample size calculation.

### Neuromuscular monitoring

Before 2017, neuromuscular transmission (NMT) monitoring was performed using Fisher & Paykel Healthcare Innervator 252 Peripheral Nerve Stimulator, TOF-Watch® and TOF-Watch SX® (Organon, Oss, The Netherlands), using qualitative and quantitative acceleromyography, respectively. An integrated NMT module using quantitative acceleromyography (Philips IntelliVue NMT module; Philips, Eindhoven, The Netherlands) was gradually introduced to Radboud UMC at the beginning of 2017. In 2017, both devices were used. Measurements taken with Innervator 252 Peripheral Nerve Stimulator and TOF-Watch (SX)® were manually entered into the anaesthesia documentation in the EMR. Therefore, all anaesthesia records were searched for a large set of NMT-related keywords, and positive results were manually checked and subsequently categorised into three distinct groups: TOFR 1.0 or more, TOFR 0.9–1.0 and TOFR < 0.9 (this includes all qualitative NMT measurements). Although these qualitative measurements might have turned out as a TOFR > 0.9 had a quantitative measurement been performed, we did not accord these manual measurements the benefit of the doubt.

All discrete data from the integrated NMT module were automatically extracted. Characteristics pertinent to the reversal of NMB by sugammadex were analysed utilising the complete data set. To discern chronological trends, the data were stratified for analyses across two temporal periods: 2015–2017 and 2018–2019. The demarcation of these time intervals was selected based on the full implementation of automatic recording in 2018, thereby facilitating an analysis of its impact on monitoring and reversal patterns.

For practical reasons, the documented timestamp of extubation might be a few minutes later than the actual moment of extubation. The NMT monitor was disconnected from the patient when TOFR was sufficiently high for extubation. However, sometimes an automated measurement occurs while the monitored arm is manipulated, resulting in an incorrect measurement. All measurements were automatically registered in the anaesthesia record. Therefore, we obtained the highest of the last five recorded NMT measurements. We considered the highest TOFR at the end of surgery for all intents and purposes the TOFR at extubation. Patients who remained intubated and were extubated at the PACU or intensive care unit (ICU) were specified as such.

### Sample size calculation and statistical analysis

The primary aim of this study was to identify factors associated with a reversal with sugammadex in surgical patients receiving NMBAs. We used multivariable logistic regression to estimate the association between patient and procedural characteristics, and sugammadex-mediated NMB reversal. A stable area under the curve (AUC) was obtained by logistic regression modelling at approximately 20–50 events per variable (van der Ploeg et al. 2014). After performing descriptive analyses of the population by time period and NMB management, a total of 14,592 adult surgical patients administered rocuronium as NMBA were included in the final analyses, resulting in a sufficient number of sugammadex events (*n* = 2111) to include over 40 variables in the regression model. With a parsimonious approach, in attempts to avoid inclusion of collinear perioperative factors in the model, we used only 14 variables (see Table 3). Data preparation and analyses were performed using SAS (version 9.4; SAS Institute, Cary, NC, USA), Microsoft Office Excel (2016), and IBM SPSS Statistics (Release 25.0.0.1).

## Results

**Fig. 1:**
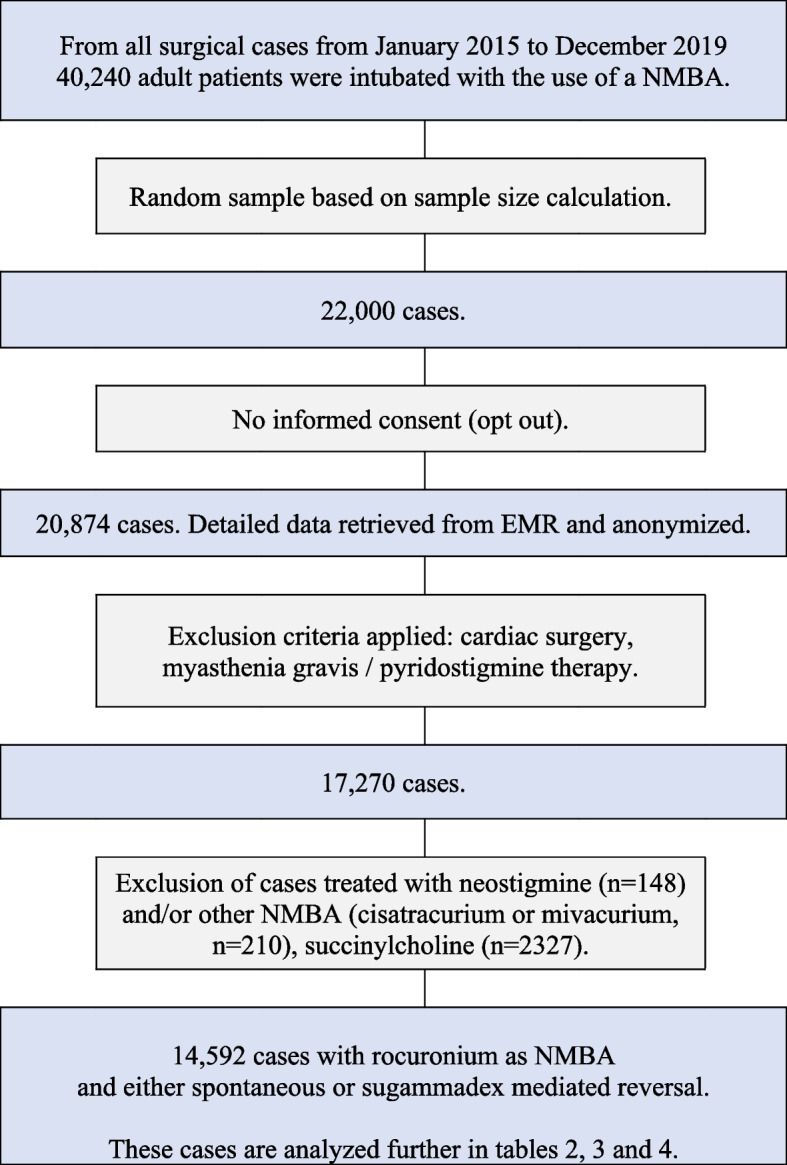
Inclusion flow-chart

In total, 20,874 cases could be used (based on opt-out consent), and data from the EMR were retrieved for analysis. All data were anonymized. Excluding cardiac surgery cases, patients diagnosed with myasthenia gravis and those receiving pyridostigmine therapy resulted in a dataset of 17,270 surgical cases (see Fig. 1). Cases treated with neostigmine (*n* = 141), other non-depolarizing NMBAs (cisatracurium and mivacurium, *n* = 210, 1%), succinylcholine alone (*n* = 2327, 13%), and ASA 5 (*n* = 3) were later excluded (ASA 5 and neostigmine due to small sample size) for the final analysis of reversal-patterns with sugammadex, resulting in a set of 14,592 cases (see Tables 1, 2 and 3).

**Table 1:** Patient characteristics per mode of reversal and time period

	2015–2017			2018–2019		
	Spontaneous recovery	Sugammadex	Total	Spontaneous recovery	Sugammadex	Total
***N***	7932 (88%)	1105 (12%)	9037 (100%)	4549 (82%)	1006 (18%)	5555 (100%)
**Sex (F)**	4116 (52%)	580 (52%)	4696 (52%)	2344 (52%)	506 (50%)	2850 (51%)
**Mean age, years (SD)**	54.0 (16.6)	56.8 (16.3)	54.4 (17.0)	53.8 (17.0)	57.6 (16.6)	54.5 (16.5)
18–39	1745 (22%)	198 (18%)	1943 (22%)	1068 (23%)	169 (17%)	1237 (22%)
40–59	2767 (35%)	339 (31%)	3106 (34%)	1508 (33%)	309 (31%)	1817 (33%)
60–69	1879 (24%)	303 (27%)	2182 (24%)	956 (21%)	247 (25%)	1203 (22%)
70–79	1219 (15%)	218 (20%)	1437 (16%)	838 (18%)	229 (23%)	1067 (19%)
80–97	322 (4%)	47 (4%)	369 (4%)	179 (4%)	52 (5%)	231 (4%)
**ASA 1**	1896 (24%)	168 (15%)	2064 (23%)	787 (17%)	83 (8%)	870 (16%)
**ASA 2**	3599 (45%)	456 (41%)	4055 (45%)	2097 (46%)	422 (42%)	2519 (45%)
**ASA 3**	1241 (16%)	227 (21%)	1468 (16%)	1061 (23%)	288 (29%)	1349 (24%)
**ASA 4**	71 (1%)	9 (1%)	80 (1%)	86 (2%)	26 (3%)	112 (2%)
**ASA not documented**	1125 (14%)	245 (22%)	1370 (15%)	518 (11%)	187 (19%)	705 (13%)
**Renal failure** ^**#**^	435 (5%)	90 (8%)	525 (6%)	227 (5%)	111 (11%)	338 (6%)
**BMI < 18.5**	199 (3%)	23 (2%)	223 (2%)	114 (2%)	18 (2%)	133 (2%)
**BMI 18.5–25**	3130 (39%)	369 (33%)	3543 (39%)	1848 (41%)	359 (36%)	2227 (40%)
**BMI 25–30**	2710 (34%)	398 (36%)	3140 (34%)	1582 (35%)	344 (34%)	1938 (35%)
**BMI 30–35**	1022 (13%)	165 (15%)	1187 (13%)	568 (12%)	144 (14%)	712 (13%)
**BMI 35–40**	293 (4%)	54 (5%)	347 (4%)	169 (4%)	52 (5%)	221 (4%)
**BMI > 40**	111 (1%)	27 (2%)	138 (2%)	60 (1%)	21 (2%)	81 (1%)

^#^ ICD diagnosis or eGFR < 30 ml/min/1.73 m^2^

**Table 2:** Case and NMT data per mode of reversal and time period

	2015–2017			2018–2019		
	Spontaneous recovery	Sugammadex	Total	Spontaneous recovery	Sugammadex	Total
**Surgical specialty**	7932 (88%)	1105 (12%)	9037 (100%)	4549 (82%)	1006 (18%)	5555 (100%)
General surgery	2049 (84%)	377 (16%)	2426 (27%)	1094 (76%)	355 (24%)	1449 (26%)
Gynecologic surgery	760 (86%)	124 (14%)	884 (10%)	387 (78%)	106 (22%)	493 (9%)
Urological surgery	765 (75%)	255 (25%)	1020 (11%)	416 (65%)	221 (35%)	637 (11%)
Lung surgery	26 (81%)	6 (19%)	32 (0%)	20 (74%)	7 (26%)	27 (0%)
Orthopaedic surgery	780 (90%)	83 (10%)	863 (10%)	412 (85%)	75 (15%)	487 (9%)
Plastic surgery	445 (93%)	31 (7%)	476 (5%)	318 (90%)	35 (10%)	353 (6%)
Neuro surgery	1093 (92%)	100 (8%)	1193 (13%)	688 (87%)	100 (13%)	788 (14%)
Ear, nose and throat surgery	1141 (94%)	68 (6%)	1209 (13%)	621 (93%)	50 (7%)	671 (12%)
Oral and maxillofacial surgery	740 (95%)	41 (5%)	781 (9%)	503 (93%)	37 (7%)	540 (10%)
Eye surgery	77 (90%)	9 (10%)	86 (1%)	29 (78%)	8 (22%)	37 (1%)
Radio therapy	16 (94%)	1 (6%)	17 (0%)	32 (97%)	1 (3%)	33 (1%)
Other specialty	40 (80%)	10 (20%)	50 (1%)	29 (73%)	11 (28%)	40 (1%)
**Open abdominal**	1419 (18%)	341 (31%)	1.760 (19%)	731 (16%)	299 (30%)	1030 (19%)
**Laparoscopic abdominal**	747 (10%)	145 (13%)	892 (10%)	315 (7%)	142 (14%)	457 (8%)
**Emergency surgery**	1194 (15%)	283 (26%)	1477 (16%)	698 (15%)	294 (29%)	992 (18%)
**Time in the OR in minutes mean; median (IQR)**	192; 155 (110; 223)	151; 131 (83; 192)	187; 152 (106; 217)	197; 166 (115; 237)	155; 129 (89; 194)	189; 158 (108; 227)
**NMT monitoring used**	3380 (43%)	846 (77%)	4226 (47%)	2590 (57%)	948 (94%)	3538 (64%)
**No (documented) NMT monitoring**	4552 (57%)	259 (23%)	4811 (53%)	1959 (43%)	58 (6%)	2017 (36%)
**PTC measured**	125 (2%)	84 (8%)	209 (2%)	100 (2%)	68 (7%)	168 (3%)
**Extubation in OR**	7058 (89%)	1062 (96%)	8120 (90%)	4210 (93%)	998 (99%)	5208 (94%)
TOFR 1.0 or more at extubation	997 (14%)	280 (26%)	1277 (16%)	1113 (26%)	386 (39%)	1499 (29%)
TOFR 0.9–1.0 at extubation	481 (7%)	111 (10%)	592 (7%)	594 (14%)	178 (18%)	772 (15%)
TOFR < 0.9 or only qualitative NMT monitor at extubation	1874 (27%)	455 (43%)	2329 (29%)	873 (21%)	384 (38%)	1257 (24%)
No NMT data in anaesthesia record	3706 (53%)	216 (20%)	3922 (48%)	1630 (39%)	50 (5%)	1680 (32%)
No NMT data in anaesthesia record, and no sugammadex			3706 (46%)			1630 (31%)
**Rocuronium total dose in mg mean; median (IQR)**	48; 40 (30; 60)	60; 50 (40; 80)	50; 40 (30; 60)	46; 40 (30; 50)	60; 50 (40; 80)	48; 40 (30; 60)
**Rocuronium total dose in ED95 equivalents (0.31 mg/kg ABW) mean; median (IQR)**	2.0; 1.7 (1.2; 2.4)	2.5; 2.0 (1.4; 3.4)	2.1; 1.7 (1.3; 2.6)	1.9; 1.7 (1.2; 2.3)	2.5; 2.2 (1.5; 3.2)	2.0; 1.7 (1.3; 2.6)
**Sugammadex total dose in mg mean; median (IQR)**		210; 200 (200; 200)			169; 200 (100; 200)	
**Sugammadex total dose in mg/kg ABW mean; median (IQR)**		2.7; 2.4 (1.9; 3.0)			2.1; 2.0 (1.3; 2.7)	
**Magnesium chloride co-administration**	365 (5%)	45 (4%)	410 (5%)	139 (3%)	35 (3%)	174 (3%)
**Use of inhalational anaesthetic**	3469 (44%)	485 (44%)	3954 (44%)	3644 (80%)	885 (88%)	4529 (82%)

**Table 3:**
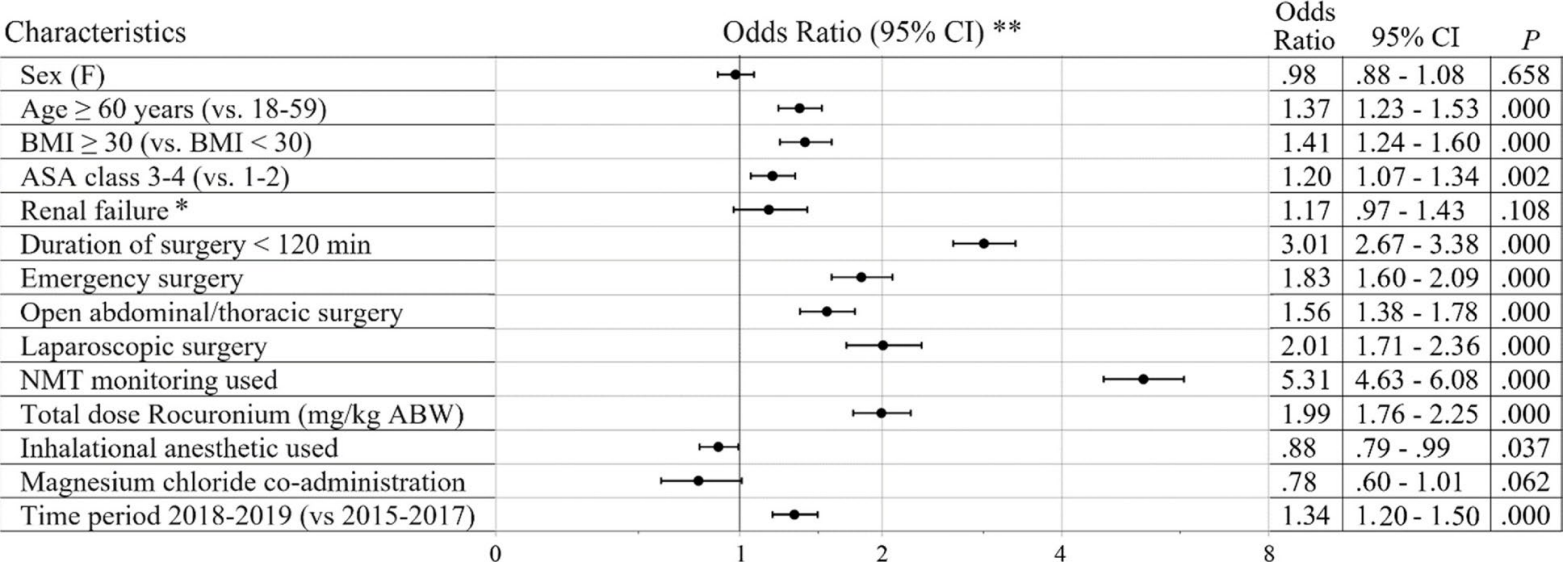
Multivariate logistic regression of characteristics associated with sugammadex reversal of NMB

* ICD diagnosis or eGFR < 30 ml/min/1.73 m^2^

** *X*-axis is on the log-scale for correct interpretation of odds ratios

### NMBAs and reversal agent (dosing) trends

In both time periods, relative NMBA usage remained the same: rocuronium (86%), suxamethonium (13%), and mivacurium or cisatracurium (1%). A total of 14,592 patients who were administered rocuronium and reversed either spontaneously or with sugammadex were included in the full analysis (Tables 1, 2 and 3). Amongst these patients, sugammadex reversal increased from 12 to 18% with a slightly lower average dose (2.7 to 2.1 mg/kg) between 2015–2017 and 2018–2019. The sugammadex reversal percentages per year were 12.8% (2015), 10.6% (2016), 13.4% (2017), 16.1% (2018) and 20.1% (2019).

The rocuronium dose was approximately the same in both time periods. In 2015–2017, the total mean dose was 50 mg (median 40; IQR 30, 60) and in ED95 equivalents 2.1 (median 1.7; IQR 1.3, 2.6). In 2018–2019, the total mean dose was 48 mg (median 40; IQR 30, 60), mean dose in ED95 equivalents 2.0 (median 1.7; IQR 1.3, 2.6). Only the spread in the spontaneously reversed group was slightly wider during 2015–2017. For sugammadex (between the two time periods assessed) the mean dose decreased from 210 mg (median 200 mg; IQR 200, 200) or 2.7 mg/kg actual body weight (ABW) (median 2.4; IQR 1.9, 3.0) to 169 mg (median 200; IQR 100, 200) or 2.1 mg/kg ABW (median 2.0; IQR 1.3, 2.7).

### Patient, surgical case and anaesthesia details (Tables 1 and 2)

Most patients were in ASA class 1 or 2 (65%). Patients with a higher ASA classification (3 or 4), older age, and serious renal disease or obesity were more often reversed with sugammadex. In some patients (~15%), mainly emergency cases, documentation of the ASA classification was omitted. About 52% of these patients were female, and no sex differences were observed in patients reversed with sugammadex. Patients were, on average, approximately 54 years old, although those actively reversed with sugammadex were slightly older (~57 years old).

General surgery was the specialty in most cases (*n* = 3.875, 27%), with the remainder spread broadly across other specialties (see Table 2). In both open (*N* = 2790, 19%) and laparoscopic (*n* = 1349, 9%) abdominal surgery, emergency surgery, and in shorter procedures, patients were more often reversed with sugammadex compared to other types of surgery. Patients with a deep NMB (as indicated by the measurement of post-tetanic count (PTC) values) were more often reversed with sugammadex. Most (~90%) patients were extubated in the operating room, although this varied somewhat by NMB reversal.

Overall, there was a total increase in (documented) NMT monitoring from 47% (2015–2017) to 64% (2018–2019) after the introduction of an integrated NMT module (Table 2). In spontaneously recovered patients, this increased from 43 to 57%, and in those reversed with sugammadex, from 77 to 94%. The percentage of patients extubated in the OR with a documented TOFR ≥ 0.9 increased from 23% (2015–2017) to 44% (2018–2019). There was a decline in patients extubated in the OR, with neither (documented) NMT monitoring nor reversal with sugammadex from 53% (2015–2017) to 39% (2018–2019).

### Logistic regression

Logistic regression analyses (Table 3) showed several patient and procedural factors independently associated with the pharmacological reversal of rocuronium-induced NMB with sugammadex. Statistically significant patient-related variables included BMI > 30 kg/m^2^ (OR, 95% CI: 1.41, 1.24–1.60), higher ASA classification (1.20, 1.07–1.34) and age > 60 years (1.37, 1.23–1.53). Surgery-related variables were even more strongly independently associated with sugammadex-mediated reversal and included duration of surgery less than 120 min (3.00, 2.67–3.38); emergency (1.83, 1.60–2.09), laparoscopic (2.00, 1.71–2.36) and open abdominal/thoracic surgery (1.56, 1.38–1.78). Associated anaesthesia-related variables included the use of NMT monitoring (5.31, 4.63–6.08), and the total dose of rocuronium corrected for ABW (1.99, 1.76–2.25). The use of inhalational anaesthetics compared to total intravenous anaesthesia was modestly, but inversely related to the use of sugammadex compared to spontaneous reversal (0.88, 0.79–.99).

## Discussion

Across the two periods studied, the distribution of patient and procedural characteristics remained relatively unchanged. However, over the last few years, we have observed a trend towards more active reversal with sugammadex. In the period 2015–2017, the sugammadex dose was more often ‘rounded off’ to a full ampule of 200 mg (67.2% compared to 56.0% of the time in 2018–2019). This could be explained by the increased use of NMT monitoring, which facilitates the detection of shallow rNMB where lower doses of sugammadex have been observed to be adequate for reliable reversal (Pongrácz et al. 2013; Schaller et al. 2010).

The POPULAR study, performed in 211 hospitals in Europe, showed the use of any NMT monitoring in only 42.1% of patients receiving a NMBA (Kirmeier et al. 2019). In this study, documented NMT monitoring in spontaneously recovered patients increased from 43 to 57% over the two consecutive periods, in those reversed with sugammadex from 77 to 94%, and overall from 47 to 64% (Table 2). A plausible explanation is the introduction of the integrated NMT module with automatic recording in the anaesthesia record, resulting in a more consistent recording compared to the manual entry of NMT data. Manual documentation can be forgotten, especially since the time around extubation is usually a relatively dynamic moment in the operating room for the anaesthesia team with many possible distractions. Therefore, the NMT monitoring data, especially from 2015 to 2017, might be an underestimation of clinical practice (i.e. what is conducted versus what is documented). Automatic registration is inherently more reliable than manual documentation, thereby minimising the potential for documentation errors. Segregating automatic and manual data for separate analyses would have resulted in overly large tables, compromising readability. Combining all data also made sense to analyse whether any NMT monitoring was used in each case.

Increasing the rate of NMT monitoring in clinical practice has proven to be a formidable challenge. Thomsen et al. (Thomsen et al. 2022) demonstrated that an e-learning module focused on NMT monitoring had no overall impact on the application of NMT monitoring, despite a post-course test suggesting an enhancement in anaesthesiologists’ knowledge within this area. Söderström et al. (Söderström et al. 2017) surveyed 653 Danish anaesthetists and found that while objective NMT monitoring is frequently utilised, it is often associated with technical difficulties. These findings are in line with our own observations and underscore the potential benefits of employing a NMT monitor that features automatic measurement and recording capabilities. Such a system is likely to enhance both the frequency and quality of these recordings, thereby diminishing the likelihood of residual neuromuscular blockade. The meticulous recording of NMT measurements is also crucial both for quality control and for medicolegal considerations. It should be noted that because the period 2015–2017 contains some automatically registered data because the integrated NMT monitor was introduced in 2017. The differences due to the automated registration of neuromuscular transmission monitoring might therefore be underestimated.

The percentage of patients extubated in the OR with neither a documented nor adequate TOFR (> 0.9) decreased from 77% in 2015–2017 to 56% in 2018–2019. This percentage of 56% is lower than the 64.7% (rNMB, defined as TOFR < 0.9) reported in the RECITE-US study years earlier (2012–2013) by Saager et al. (Saager et al. 2019). When comparing our data directly to prospective studies measuring rNMB in the PACU, it is important to realise that not all of the patients in our study, where no (quantitative) NMT monitoring was used or documented, had rNMB. Nevertheless, when (quantitative) NMT monitoring is not performed in patients who receive an NMBA, there is a certain risk of rNMB (Murphy et al. 2011). In some cases, no (further) NMT measurements were carried out after the administration of sugammadex, with only a small chance of rNMB due to the predictable nature of sugammadex-mediated reversal. Therefore, amongst patients reversed with sugammadex, the last TOFR measured prior to extubation may not be distinguishable from the TOFR measured prior to the administration of sugammadex and is therefore not easily compared to rNMB risk amongst spontaneously reversed patients. Although the administration of sugammadex without NMT monitoring does not completely eliminate the risk of rNMB (Kotake et al. 2013; Nemes et al. 2017) it decreases the risk significantly. Patients extubated in the OR, with spontaneous recovery of NMB, with no documented NMT monitoring at all, or an inadequate TOFR at extubation, are obviously most at risk for complications due to rNMB. These percentages decreased from 46% (spontaneous recovery with no documented NMT) and 23% (spontaneous recovery with inadequate TOFR at extubation) during 2015–2017 to 31% and 17% during 2018–2019, respectively. Continuing NMT monitoring until full recovery from NMB after sugammadex reversal could further decrease the risk of rNMB (Kotake et al. 2013).

Logistic regression analysis (Table 3) revealed independent factors associated with the pharmacological reversal of rocuronium-induced neuromuscular blockade with sugammadex. Patient-related variables included high BMI, higher ASA classification, and age > 60 years. This may be attributed to a more careful approach of anaesthesiologists regarding the prevention of rNMB in patients with a higher risk of perioperative complications, including those with a higher ASA classification, age, or BMI. This is in concordance with data from the Multicentre Perioperative Outcomes Group in the United States published by Dubovoy et al. (Dubovoy et al. 2020) and data from Leiden University Medical Centre (another large teaching hospital in the Netherlands) presented by Martini et al. (Martini et al. 2021).

Surgery-related factors associated with the use of sugammadex are the duration of surgery less than 120 min, and emergency, laparoscopic, and open abdominal/thoracic surgery. Martini et al. also found an association between reversal with sugammadex and shorter duration of surgery (Martini et al. 2021). In both open abdominal or thoracic surgery and laparo/thoracoscopic procedures, NMBAs are often repeatedly administered to improve surgical conditions until the end of surgery. Additionally, open abdominal or thoracic surgery is inherently associated with a higher risk of pulmonary complications (Canet et al. 2015) and rNMB might therefore be treated more rigorously. Patients who underwent emergency surgery were more often reversed with sugammadex. This can be readily explained by the fact that many of these patients were intubated following rapid sequence induction of anaesthesia with a high dose of rocuronium. A higher dose of rocuronium resulted in a higher chance of rNMB and a need for active reversal with sugammadex. To illustrate, in 30.3% of emergency cases where rocuronium was used, the total dosage of rocuronium was at least 3× ED95 vs 20.6% for non-emergency surgery. One thousand seventy patients with an emergency case received succinylcholine as the only NMBA, but these were excluded from the final analysis.

Anaesthesia-related factors associated with the use of sugammadex included the use of NMT monitoring and the total dose of rocuronium corrected for ABW. When NMT monitoring is used, there is a higher chance of diagnosing rNMB. However, the observed relationship with sugammadex administration may also be explained by our local practice, as in our hospital, sugammadex is rarely administered without a NMT measurement. NMT monitoring will also be used more often when there is a high chance of rNMB due to a recent rocuronium dose or high clinical suspicion of rNMB. This is in concordance with the fact that NMT monitoring is highly correlated with sugammadex-mediated reversal.

When a higher intraoperative total dose of rocuronium is used, there may also be a higher risk of rNMB. This was reflected in the results. We used a variable that was corrected for ABW (ED95) to better compare patients.

Both magnesium chloride (Fuchs-Buder et al. 1995) and inhalational anaesthetics (Jellish et al. 2000; Wulf et al. 1998) are known to increase the potency of NMBA. Sevoflurane and isoflurane are the only inhalational anaesthetics used at our hospital. There was a slightly lower chance of sugammadex reversal in patients receiving inhalation anaesthetics for maintenance of anaesthesia (odds ratio 0.883, 95% CI 0.785–0.992). A possible explanation for this observation could be that, in general, lower doses of rocuronium are required to achieve the same level of muscle relaxation during inhalation anaesthesia. Magnesium chloride is mainly used as a multimodal analgesic in our hospital because of its N-methyl-D-aspartate (NMDA) receptor-blocking properties. Our analysis did not reveal a significant association between sugammadex reversal and magnesium chloride use.

### Strengths and limitations

A strength of this study is that the combination of automatic extraction from the EMR and manual checks ensures high-quality data and a large dataset with sufficient power for analysis of NMBA and sugammadex dosing and NMT monitoring. Because this is a single-centre study in a tertiary teaching hospital, the results are likely more generalizable to other local tertiary care centres than to community or smaller nonteaching hospitals. These hospitals often have a higher turnover of shorter procedures, and pharmacological reversal may be used more often. A study by Dubovoy et al. (Dubovoy et al. 2020) showed a wide variation in sugammadex use amongst different hospitals in the US. However, in the absence of global guidelines on NMB management, there is a large variation in NMB management, specifically reversal trends between regions, reflecting variability in practice, training, and exposure to new technologies.

## Conclusion

There were no clear changes in NMBA types or dosage in our dataset between 2015 and 2019, but there was a trend towards more active reversal with a more precise dose of sugammadex. In patients with a presumed higher a priori risk of pulmonary complications (i.e. high age, high ASA score, high BMI, abdominal or thoracic surgery), reversal with sugammadex is more frequently used. In addition, the use of higher doses of rocuronium (e.g. deep NMB or emergency surgery) is associated with the use of sugammadex. Our data indicate that the implementation of NMT monitoring with automatic recording may have contributed to a reduction in the number of patients extubated in the OR, with spontaneous recovery, without documented NMT measurements, or with an inadequate TOFR at extubation.

## Data Availability

The datasets used and/or analysed during the current study are available from the corresponding author on reasonable request.

## References

[CR1] Berg H, Roed J, Viby-Mogensen J, Mortensen CR, Engbaek J, Skovgaard LT, Krintel JJ (1997). Residual neuromuscular block is a risk factor for postoperative pulmonary complications. A prospective, randomised, and blinded study of postoperative pulmonary complications after atracurium, vecuronium and pancuronium. Acta Anaesthesiol Scand.

[CR2] Blobner M, Hunter JM, Meistelman C, Hoeft A, Hollmann MW, Kirmeier E, Lewald H, Ulm K (2020). Use of a train-of-four ratio of 0.95 versus 0.9 for tracheal extubation: an exploratory analysis of POPULAR data. Br J Anaesth.

[CR3] Bowman WC (2006). Neuromuscular block. Br J Pharmacol.

[CR4] Bruintjes MH, van Helden EV, Braat AE, Dahan A, Scheffer GJ, van Laarhoven CJ, Warlé MC (2017). Deep neuromuscular block to optimize surgical space conditions during laparoscopic surgery: a systematic review and meta-analysis. Br J Anaesth.

[CR5] Cammu G. Residual Neuromuscular Blockade and Postoperative Pulmonary Complications: What Does the Recent Evidence Demonstrate? Curr Anesthesiol Rep. 2020:1–6. 10.1007/s40140-020-00388-4 PMC7222856 32421054

[CR6] Canet J, Sabaté S, Mazo V, Gallart L, de Abreu MG, Belda J, Langeron O, Hoeft A, Pelosi P (2015). Development and validation of a score to predict postoperative respiratory failure in a multicentre European cohort: A prospective, observational study. Eur J Anaesthesiol.

[CR7] Dubovoy TZ, Saager L, Shah NJ, Colquhoun DA, Mathis MR, Kapeles S, Mentz G, Kheterpal S, Vaughn MT (2020). Utilization Patterns of Perioperative Neuromuscular Blockade Reversal in the United States: A Retrospective Observational Study From the Multicenter Perioperative Outcomes Group. Anesth Analg.

[CR8] Eikermann M, Groeben H, Hüsing J, Peters J (2003). Accelerometry of adductor pollicis muscle predicts recovery of respiratory function from neuromuscular blockade. Anesthesiology.

[CR9] Eikermann M, Vogt FM, Herbstreit F, Vahid-Dastgerdi M, Zenge MO, Ochterbeck C, de Greiff A, Peters J (2007). The predisposition to inspiratory upper airway collapse during partial neuromuscular blockade. Am J Respir Crit Care Med.

[CR10] Fuchs-Buder T, Wilder-Smith OH, Borgeat A, Tassonyi E (1995). Interaction of magnesium sulphate with vecuronium-induced neuromuscular block. Br J Anaesth.

[CR11] Grabitz SD, Rajaratnam N, Chhagani K, Thevathasan T, Teja BJ, Deng H, Eikermann M, Kelly BJ (2019). The Effects of Postoperative Residual Neuromuscular Blockade on Hospital Costs and Intensive Care Unit Admission: A Population-Based Cohort Study. Anesth Analg.

[CR12] Jellish WS, Brody M, Sawicki K, Slogoff S (2000). Recovery from neuromuscular blockade after either bolus and prolonged infusions of cisatracurium or rocuronium using either isoflurane or propofol-based anesthetics. Anesth Analg.

[CR13] Kirmeier E, Eriksson LI, Lewald H, Jonsson Fagerlund M, Hoeft A, Hollmann M, Meistelman C, Hunter JM, Ulm K, Blobner M (2019). Post-anaesthesia pulmonary complications after use of muscle relaxants (POPULAR): a multicentre, prospective observational study. Lancet Respir Med.

[CR14] Kotake Y, Ochiai R, Suzuki T, Ogawa S, Takagi S, Ozaki M, Nakatsuka I, Takeda J (2013). Reversal with sugammadex in the absence of monitoring did not preclude residual neuromuscular block. Anesth Analg.

[CR15] Martini C, Boon M, Olofsen E, Bash L, Dahan A (2021). Determinants for reversal versus spontaneous recovery of neuromuscular blockade following general anesthesia in a university center in the Netherlands: a retrospective observational study.

[CR16] Murphy GS, Szokol JW, Avram MJ, Greenberg SB, Marymont JH, Vender JS, Gray J, Landry E, Gupta DK (2011). Intraoperative acceleromyography monitoring reduces symptoms of muscle weakness and improves quality of recovery in the early postoperative period. Anesthesiology.

[CR17] Nemes R, Fulesdi B, Pongracz A, Asztalos L, Szabo-Maak Z, Lengyel S, Tassonyi E (2017). Impact of reversal strategies on the incidence of postoperative residual paralysis after rocuronium relaxation without neuromuscular monitoring: A partially randomised placebo controlled trial. Eur J Anaesthesiol.

[CR18] Pongrácz A, Szatmári S, Nemes R, Fülesdi B, Tassonyi E (2013). Reversal of neuromuscular blockade with sugammadex at the reappearance of four twitches to train-of-four stimulation. Anesthesiology.

[CR19] Saager L, Maiese EM, Bash LD, Meyer TA, Minkowitz H, Groudine S, Philip BK, Tanaka P, Gan TJ, Rodriguez-Blanco Y (2019). Incidence, risk factors, and consequences of residual neuromuscular block in the United States: The prospective, observational, multicenter RECITE-US study. J Clin Anesth.

[CR20] Schaller SJ, Fink H, Ulm K, Blobner M (2010). Sugammadex and neostigmine dose-finding study for reversal of shallow residual neuromuscular block. Anesthesiology.

[CR21] Söderström CM, Eskildsen KZ, Gätke MR, Staehr-Rye AK (2017). Objective neuromuscular monitoring of neuromuscular blockade in Denmark: an online-based survey of current practice. Acta Anaesthesiol Scand.

[CR22] Thomsen JLD, Mathiesen O, Hägi-Pedersen D, Skovgaard LT, Østergaard D, Gätke MR (2022). Improving neuromuscular monitoring and reducing residual neuromuscular blockade via e-learning: A multicentre interrupted time-series study (INVERT study). Acta Anaesthesiol Scand.

[CR23] van der Ploeg T, Austin PC, Steyerberg EW (2014). Modern modelling techniques are data hungry: a simulation study for predicting dichotomous endpoints. BMC Med Res Methodol.

[CR24] Viby-Mogensen J (2000). Postoperative residual curarization and evidence-based anaesthesia. Br J Anaesth.

[CR25] Viby-Mogensen J, Engbaek J, Eriksson LI, Gramstad L, Jensen E, Jensen FS, Koscielniak-Nielsen Z, Skovgaard LT, Ostergaard D (1996). Good clinical research practice (GCRP) in pharmacodynamic studies of neuromuscular blocking agents. Acta Anaesthesiol Scand.

[CR26] Viby-Mogensen J, Jørgensen BC, Ording H (1979). Residual curarization in the recovery room. Anesthesiology.

[CR27] Watts RW, London JA, van Wijk RM, Lui YL (2012). The influence of unrestricted use of sugammadex on clinical anaesthetic practice in a tertiary teaching hospital. Anaesth Intensive Care.

[CR28] Wulf H, Ledowski T, Linstedt U, Proppe D, Sitzlack D (1998). Neuromuscular blocking effects of rocuronium during desflurane, isoflurane, and sevoflurane anaesthesia. Can J Anaesth.

[CR29] Xará D, Santos A, Abelha F (2015). Adverse respiratory events in a post-anesthesia care unit. Arch Bronconeumol.

